# Connaissances, attitudes et pratiques des parents face à la vaccination contre la poliomyélite à Abéché-Tchad

**DOI:** 10.11604/pamj.2018.31.219.12966

**Published:** 2018-12-04

**Authors:** Abderahim Mahamat Nadjib, Harvey Attoh-Touré, Adam Abdel-mahamoud, Sabine Baron, Solène Brunet-Houdard, Emmanuel Rusch, Leslie Grammatico-Guillon

**Affiliations:** 1 Institut National Supérieur des Sciences et Techniques d’Abéché, Tchad; 2 Laboratoire de Santé Publique, EE1 EES, Université François Rabelais, Tours, France; 3 Département de Santé Publique, Université F Houphouët-Boigny d’Abidjan, Côte d’Ivoire; 4 SIMEES, Unité Régionale d'Epidémiologie Hospitalière, CHRU de Tours; 5 SIMEES, Unité d'Évaluation Médico-Economique, CHRU de Tours; 6 Faculté de Médecine, Université François Rabelais, Tours, France

**Keywords:** Vaccination, poliomyélite, enquête, ménages, parents, Abéché, Immunization, poliomyelitis, investigation, households, parents, Abéché

## Abstract

**Introduction:**

au Tchad, la transmission de la poliomyélite a été interrompue en 2000, mais les importations à partir du Nigéria et la faiblesse des couvertures vaccinales constituent un risque majeur de relance de la maladie. L'objectif de ce travail était d'analyser les connaissances, attitudes et pratiques des parents vis-à-vis de la vaccination contre la poliomyélite des enfants âgés de 0 à 5 ans au Tchad.

**Méthodes:**

cette étude transversale a été réalisée dans les six arrondissements d'Abéché. Seuls les ménages qui avaient des enfants de moins de 5 ans ont été inclus. Les données ont été recueillies par des entretiens avec les parents et tuteurs d'enfants éligibles à l'aide d'un questionnaire testé et validé.

**Résultats:**

nous avons interrogé 210 ménages. Aucune famille ne possédait de carnet de vaccination de leurs enfants. Cependant, 97% ont déclaré avoir des enfants ayant participé aux campagnes de vaccination de masse. Près de 97% connaissaient la poliomyélite et 98% avaient entendu parler des campagnes de vaccination. Les canaux d'information les plus cités étaient la radio (98%) et les agents vaccinateurs (72%). Seul 3% des parents interrogés ont déclaré avoir refusé la vaccination. Il existait une association entre l'influence négative de l'entourage et la non vaccination des enfants (p = 0,005).

**Conclusion:**

les connaissances sur la maladie et le vaccin sont bonnes au Tchad malgré l'existence de rumeurs concernant notamment les effets des vaccins. L'absence de carnet de vaccination a limité l'analyse des résultats de l'enquête, uniquement déclarative avec un taux de vaccination très élevé déclaré. Dans le cadre de l'éradication, le carnet est indispensable pour accompagner la politique de prévention.

## Abstract (en)

**Introduction:**

in Chad, transmission of poliovirus has been interrupted in 2000, but imports from Nigeria and weakness of vaccination coverage are a major risk of disease reactivation. This study aims to investigate knowledge, attitudes and practices of parents of children aged 0 to 5 years on vaccination against polio in Chad.

**Methods:**

this cross-sectional study was carried out in the six districts of Abéché. Only households who had children under 5 years of age were included. Data were collected through interviews with parents and guardians of eligible children using a tested and validated questionnaire.

**Results:**

we interviewed 210 households. No family had a vaccination record notebook of their children. However, 97% reported vaccinated children who had participated in mass vaccination campaigns. About 97% were aware of poliomyelitis disease and 98% knew vaccination campaign. The most cited channels of information were radio (98%) and vaccinators (72%). Only 3% of parents reported refusing vaccination. There was an association between the negative influence of the relatives and the non-vaccination of children (p = 0.005).

**Conclusion:**

disease and vaccine knowledge is good in Chad despite the existence of rumours about, in particular, vaccine effects. The lack of immunization cards limited the analysis of survey results which were only declarative with a very high declared vaccination rate. Immunization cards are essential for eradication in association with prevention policy.

## Introduction

L'Initiative Mondiale pour l'Eradication de la Poliomyélite (IMEP) a été lancée en 1988 par l'Assemblée Mondiale de la Santé afin d'interrompre la transmission du Poliovirus Sauvage (PVS) [1, 2]. De 1988 à 2002, l'ensemble des pays développés a réussi à éradiquer la poliomyélite et de nombreux pays en développement ont été déclarés indemnes de poliomyélite [3]. Toutefois, malgré ces avancées, les efforts d'éradication de la poliomyélite se sont heurtés à des difficultés notamment l'importation de PVS et/ou de souches vaccinales (PVDVc2), des pays endémiques (Afghanistan, Pakistan et Nigéria) vers les pays exempts de poliomyélite [4] entrainant un recul de la date attendue d'éradication mondiale de 2012 à 2018 [1, 5]. La persistance de l'endémie au Nigéria expose le Tchad et les autres pays voisins à un risque de nouvelles importations. Au Tchad, la transmission de la poliomyélite a été interrompue en 2000. Cependant, la faiblesse des couvertures vaccinales estimée à 31% pour la 3^ème^ dose de vaccination antipoliomyélitique oral en 2011 ainsi que les importations à partir du Nigéria voisin ont entraîné un rétablissement de la circulation du PVS [2, 6, 7]. Ainsi, en 2011, 132 cas de poliomyélite ont été confirmés au Tchad, qui a également notifié entre juillet et août 2012 cinq cas de poliomyélite provoqués par un Poliovirus dérivé de la souche vaccinale [2, 7].

Le Tchad est toujours confronté à de nombreux défis dans son objectif d'éradication de la poliomyélite. Le manque de ressources humaines et financières, l'insuffisance de la surveillance épidémiologique et environnementale, le manque d'information et de sensibilisation entraînant la non-adhésion des populations constituent les freins majeurs à l'augmentation de la couverture vaccinale des enfants [7]. Malgré tout, le Tchad poursuit ses efforts par le renforcement de la surveillance et par des activités de vaccination systématique et supplémentaire [2]. Les recommandations vaccinales actuelles sont que chaque enfant reçoive au moins 3 doses de vaccin poliomyélite oral. Dans ce contexte, l'adhésion des parents joue un rôle important. L'objectif de ce travail était d'analyser chez les parents des enfants âgés de 0 à 5 ans les connaissances, attitudes et pratiques vis-à-vis de la vaccination contre la poliomyélite à Abéché. Les objectifs spécifiques visaient à: décrire les connaissances et attitudes des parents sur la vaccination contre la poliomyélite; décrire les pratiques en matière de vaccination contre la poliomyélite; Identifier les motifs de refus de la vaccination contre la poliomyélite.

## Méthodes

**Type de l'étude:** il s'agissait d'une étude transversale à visée descriptive concernant les connaissances, attitudes et pratiques des parents d'enfants de 0 à 5 ans vis-à-vis de la vaccination contre la poliomyélite.

**Figure 1:**
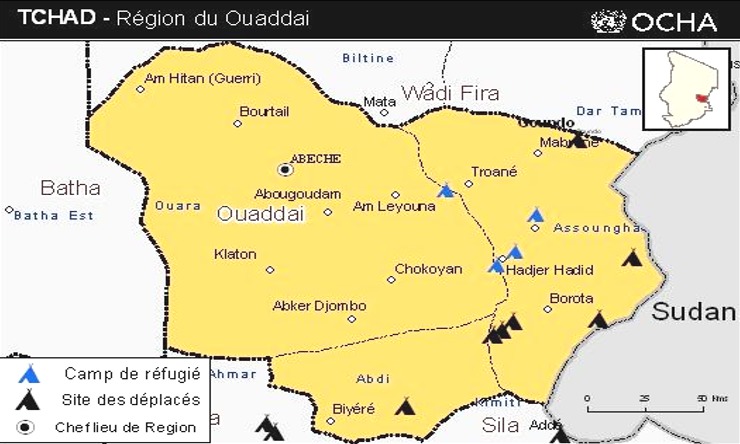
carte administrative de la région du Ouaddaï

**Cadre de l'étude:** l'étude s'est déroulée dans la ville d'Abéché (chef-lieu simultanément de la Région du Ouaddaî et celui du Département de Ouara). Elle est subdivisée en six (6) arrondissements regroupant environ quarante-six (46) quartiers [8] (138 684 habitants) [9]. Elle est située à environ 900 km de N'Djamena, capitale du Tchad. Abéché est la plus grande ville de l'Est et la troisième au niveau national après N'Djamena et Moundou [8] (Figure 1). A proximité de la ville, se trouve le camp de Gaga (zone de responsabilité du district d'Abéché) avec 18 000 réfugiés Soudanais [10].

**Période de l'étude:** la collecte de données s'est déroulée du 10 janvier au 10 avril 2016 dans les six arrondissements de la ville d'Abéché.

**Population d'étude:** nous avons inclus un échantillon de foyers, mères, pères et tuteurs d'enfants de 0 à 5 ans, résidant dans la zone d'étude au moment de l'enquête et qui ont accepté d'y participer. Ils ont été sélectionnés de manière aléatoire dans les quartiers de la ville d'Abéché. La méthode de sélection de notre échantillon s'est basée sur les enquêtes de couverture vaccinale par sondage en grappe à 2 degrés type OMS [11]. Le nombre de sujet nécessaire a été obtenu à partir de la formule suivante:

$$\text{N}=\text{e}\left({\text{Zα}}^{\text{2*}}{\text{pq/i}}^{2}\right)$$

**Figure 2:**
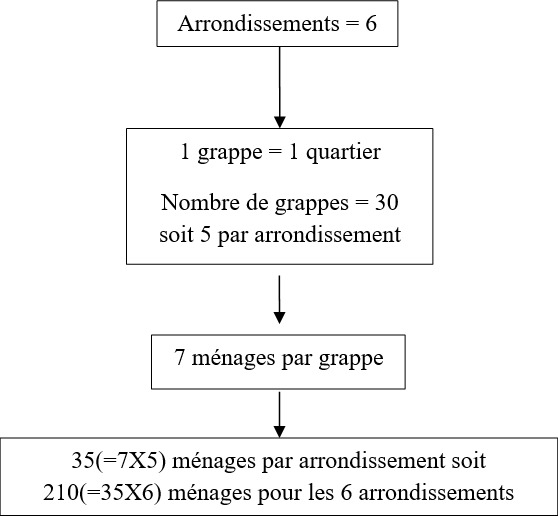
méthode d’échantillonnage

N = taille de l'échantillon; e = effet de grappe (estimé à 2, avec cette méthode de sondage); Zα = 1,96 pour un risque d'erreur α = 0,05; p = taux attendu de 0,5(50%) et q = 1-p = 0,50; i= précision fixée pour cette étude = 10% Ainsi, cette méthode nous a amenés à enquêter 210 ménages repartis uniformément dans 30 grappes tirées au hasard à raison de sept ménages par grappe. Les grappes dont l'unité était le quartier ont été tirés au sort sur la base de la liste complète des quartiers qui composaient les 6 (six) arrondissements de la ville d'Abéché (Figure 2). Devant l'absence d'une base de sondage des ménages, nous avons sélectionné les ménages de proche en proche. A partir d'un croisement situé au centre de chaque quartier, l'enquêteur a choisi une direction au hasard: il a ensuite jeté en l'air un stylo dont la pointe à servi à indiquer la direction à suivre. En suivant cette direction, il a procédé à la sélection des ménages. La première maison à droite était la première à visiter. S'il n'y avait aucune maison à droite, il choisissait le côté gauche. En cas d'absence d'enfants de 0 à 5 ans ou de refus de participation, l'enquêteur devait passer au ménage suivant, de proche en proche jusqu'à obtenir le nombre requis.

**Collecte des données:** la variable d'intérêt « vaccination contre la poliomyélite des enfants du ménage » était collectée de façon déclarative auprès du parent participant à l'étude en variable binaire « fratrie ayant participé aux campagnes de vaccination de masse » (oui/non). Un questionnaire sur la vaccination contre la poliomyélite a été construit et un prétest a été réalisé sur 12 parents choisis au hasard. Ceci nous a permis de corriger et valider notre outil de collecte des données. La collecte a été effectuée par des entretiens en face à face. Ces entretiens étaient effectués en français ou en langue locale (ce qui était le plus souvent nécessaire). Ils ont été réalisés par le doctorant et deux enquêteurs préalablement formés. En effet, la formation consistait à promouvoir une attitude neutre des enquêteurs vis-à-vis de la vaccination afin d'obtenir des réponses sincères des répondants, notamment sur le statut vaccinal déclaré de la fratrie. L'attention était portée sur le fait de ne pas être confondu avec les agents superviseurs des Journées Nationales de Vaccination. Les variables collectées étaient: caractéristiques sociodémographiques des personnes interrogées (sexe, lieu de résidence, âge, statut matrimonial, nombre d'enfants, lien parental avec l'enfant, profession, religion, niveau d'étude); connaissances (de la maladie, du vaccin, des campagnes de vaccination); attitudes (acceptation ou refus du vaccin, influence de l'entourage); pratiques vis-à-vis de la vaccination contre la poliomyélite: en l'absence de carnet de vaccination dans les ménages visités, le statut vaccinal des enfants d'un ménage a été estimé par la participation ou non des enfants aux campagnes de vaccination de masse contre la poliomyélite; réticence à la vaccination contre la poliomyélite.

**Analyse des données:** les données ont été collectées et analysées avec le logiciel EPI INFO version 2007. Les pourcentages ont été comparés par un test du Chi deux et/ou test exact de Fisher avec un seuil de signification à 0,05.

**Considérations éthiques:** avant de commencer l'enquête, nous avons obtenu une autorisation administrative de la part du Ministère de la Santé Publique. Les chefs d'arrondissements et les chefs des quartiers ont été impliqués dans cette démarche. Les participants ont été informés du caractère anonyme de l'enquête et ont donné préalablement leur consentement verbal.

## Résultats

Parmi les 210 ménages enquêtés, 97% ont déclaré des enfants ayant participé aux campagnes de vaccination de masse.

**Caractéristiques sociodémographiques des parents enquêtés:** les femmes représentaient 66,7% des répondants de l'étude avec 40% de ménagères âgées de 26 à 35 ans (40,5%). La majorité des répondants ont déclaré être mariés (81,4%) et de confession musulmane (85,2 %). Enfin, 38% des participants n'avaient pas été scolarisés et 91% des foyers déclaraient avoir entre 1 et 3 enfants.

**Caractéristiques des parents d'enfants n'ayant pas participé aux campagnes de vaccination de masse (N = 6):** seuls 3% des parents ont déclaré que leurs enfants n'avaient pas participé aux campagnes de vaccination de masse contre la poliomyélite. Il s'agissait de quatre couples avec 2 enfants, et deux couples avec des fratries de trois. Les caractéristiques sociodémographiques de ces parents étaient les suivants: leur âge variait de 26 à 45 ans, et ils déclaraient être de confession musulmane et mariés (Tableau 1); sex-ratio = 1 (parents interrogés); on retrouvait 3 hommes, commerçants, avec un niveau scolaire primaire et 3 femmes, ménagères et non scolarisées (Tableau 1). Ils ont tous déclaré que le vaccin était nuisible et provoquait des effets secondaires pour les enfants. Ils déclaraient avoir subi également l'influence négative de leur entourage.

**Tableau 1:** caractéristiques sociodémographiques de la population d’étude (N = 210)

Caractéristiques des parents	Effectif	Pourcentage (%)
**Tranche d’âge**		
18-25	71	33,8
26-35	85	40,5
36-45	34	16,2
46-55	14	6,6
56-65	6	2,9
**Sexe**		
Homme	70	33,3
Femme	140	66,7
**Statut matrimonial**		
Célibataire	4	1,9
Marié	171	81,4
Divorcé	11	5,3
Veuf (ve)	24	11,4
**Nombre d’enfants (de 0 à 5 ans)**		
0 enfant	3	1,4
1 enfant	57	27,1
2 enfants	89	42,4
3 enfants	46	21,9
4 enfants	15	7,2
**Lien parental**		
Père	65	30,9
Mère	136	64,8
Autre	9	4,3
**Profession**		
Ménagère	84	40,0
Commerçant	52	24,8
Elève/étudiant	26	12,4
Fonctionnaire	16	7,6
Ouvrier	19	9,0
Agriculteur	9	4,3
Artisan	4	1,9
**Niveau d’instruction**		
Ecole coranique	33	15,7
Non scolaire	79	37,6
Primaire	32	15,3
Secondaire	26	12,4
Supérieur	40	19,0
**Religion**		
Islam	179	85,2
Christianisme	31	14,8

**Connaissances, attitudes et pratiques des parents vis-à-vis de la vaccination contre la poliomyélite:** les participants pouvaient choisir plusieurs items dans le questionnaire. Plus de 97% ont déclaré connaître la poliomyélite et 63% ont reconnu qu'il s'agissait d'une maladie invalidante (Tableau 2), sans traitement possible (64%). Ils savaient que le vaccin était oral, et non nuisible pour l'enfant. Toutefois 24% évoquaient la possibilité d'effets secondaires. Parmi les parents/tuteurs, 98,1% avaient entendu parler des campagnes de vaccination. Les 85% des répondants ont été suffisamment informés des campagnes de vaccination et 86% avaient compris l'enjeu. Les canaux d'information les plus cités étaient la radio locale (97,6%) et les agents vaccinateurs (72,4%). La vaccination a eu lieu au domicile pour 99% des répondants (Tableau 3). Malgré la relativement bonne connaissance de la population d'étude de la maladie et du vaccin plus de la moitié (61%) des parents avaient déclaré avoir subi l'influence négative de leur entourage. Parmi les participants déclarant avoir déjà vacciné leurs enfants, 9,5% auraient refusé le vaccin dans une campagne précédente et pourraient refuser le vaccin lors d'une campagne future. La participation des enfants aux campagnes de masse était moindre dans les ménages qui considéraient le vaccin nuisible. La connaissance des effets secondaires influençait également négativement la participation aux campagnes de vaccination de masse (Tableau 4). Les répondants qui habitaient dans le 2^e^, 3^e^ et 5^e^ arrondissement subissaient plus l'influence négative de leur entourage (Tableau 5).

**Tableau 2:** répartition des enquêtés et de leur taux de participation selon leur connaissance de la maladie et du vaccin

Connaissances des parents	Effectif	Pourcentage (%)
**Connaissez- vous la poliomyélite? N= 210**		
Oui	203	96,7
Non	7	3,3
**La poliomyélite est une maladie**		
Invalidante	130	61,9
Surnaturelle	106	50,5
Héréditaire	12	5,7
Maladie du sang	9	4,3
Autre	3	1,4
**Existe-t-il des remèdes pour la poliomyélite? N= 210**		
Oui	75	35,7
Non	135	64,3
**Si oui, le ou lesquels? N = 75**		
Hôpital	13	17,3
Invocation Dieu	24	32,0
Tradipraticien	26	34,7
Vaccination	12	16,0
**Le vaccin contre la polio est?**		
Liquide : deux Gouttes	206	98,1
Injection	11	5,2
Autre	3	1,4
**L’objectif du vaccin est**		
Guérir	74	35,2
Protéger	177	84,3
Autre	1	0,5
**Est- il nuisible pour les enfants? N = 210**		
Oui	19	9,0
Non	191	91,0
**Connaissance des effets secondaires N = 210**		
Oui	50	23,8
Non	160	76,2

**Tableau 3:** répartition des enquêtés selon leur connaissance des campagnes de vaccination

Connaissances des parents	Effectif	Pourcentage (%)
**Entendu parler des campagnes**		
**N = 210**		
Oui	206	98,1
Non	4	1,9
**Canal d’information**		
Affiche	32	15,2
Agent santé	70	33,3
Agent vaccinateur	152	72,4
Banderole	20	9,5
Crieur public	16	7,6
Eglise	24	11,4
Journaux	6	2,9
Leader opinion	33	15,7
Radio	205	97,6
Télé	70	33,3
**Suffisamment informé N = 210**		
Oui	179	85,2
Non	31	14,8
**Bonne compréhension de l'information**		
**N = 210**		
Oui	181	86,2
Non	29	13,8

**Tableau 4:** participation des enquêtés selon leurs croyances, connaissances et influence de l’entourage sur le vaccin poliomyélite (N = 210)

Questions posées aux participants N = 210	Enfants ayant participés aux campagnes	Enfants n’ayant pas participés aux campagnes	RR	IC	P
**Est-il nuisible pour les enfants ?**					
Nuisible	13	6	0,68	0,5-0,93	P < 10^-3^
Non Nuisible	191	0			
**Influence négative de l’entourage ?**					
Influence négative	122	6	0,95	0,91-1	NS
Pas d’influence négative	82	0			
**Connaissez-vous les effets secondaires ?**					
Connait	44	6	0,88	0,79-0,97	P <10^-3^
Ne connait pas	160	0			

**Tableau 5:** répartition des enquêtés selon l’influence négative de l’entourage par arrondissement

Arrondissement	Pas d'influence	Influence négative	P
Premier arrondissement	18	17	0.03
Deuxième arrondissement	9	26	
Troisième arrondissement	9	26	
Quatrième arrondissement	17	18	
Cinquième arrondissement	9	26	
Sixième arrondissement	17	18	
**Total**	**82**	**128**	

## Discussion

Aucune étude similaire portant sur les connaissances, attitudes et pratiques des parents d'enfants de 0 à 5 ans vis-à-vis de la vaccination contre la poliomyélite n'a été effectuée au Tchad. Il existe quelques études sur les flambées de poliomyélite et le progrès vers l'éradication [2, 6]. La plupart des parents interviewés avaient des connaissances sur la poliomyélite et une attitude positive envers la vaccination. Près de 98% avaient déclaré avoir des enfants ayant participé aux campagnes de vaccination. Une étude menée par Khan en 2015 au Pakistan, pays endémique avait noté 84,8% d'opinions défavorables à la vaccination [12]. La raison probable de cette différence pourrait être due à l'intensification des campagnes de vaccination organisées au Tchad depuis 2011 pour stopper les flambées dues aux importations. En outre, la mobilisation et l'implication des leaders locaux [2, 6] auraient joué un rôle positif dans l'acceptation de la vaccination. Cependant, le type d'enquête par questionnaire face à face a pu biaiser le recueil de la variable d'intérêt avec surestimation de la couverture vaccinale probable (questionnaire déclaratif sur la vaccination). Les enquêteurs étaient formés pour être les plus neutres possibles pour recueillir les réponses et se différencier des agents superviseurs lors des journées de vaccination nationales. Il n'y a malheureusement pas de carnet de vaccination ou autre document attestant la vaccination au Tchad.

Les femmes (66,7%), notamment celles âgées de 18 à 45 ans (90, 5%) étaient les principales accompagnatrices des enfants. Ces résultats sont semblables à ceux d'une étude similaire réalisée au Nigéria par Murele et collaborateurs (en 2014), où 66% des accompagnatrices étaient des femmes âgées de 18 à 45 ans [13]. Cela pourrait s'expliquer par le fait qu'au sein de la famille dans la civilisation traditionnelle africaine, les femmes ont pour mission de prodiguer des soins à toute la famille tandis que les hommes doivent se soucier des besoins financiers et matériels [14]. De ce fait, peu d'hommes ont participé à notre enquête car probablement au travail. Fort de ce constat, les enquêteurs avaient revu les horaires de passage (tôt le matin ou en fin de l'après midi) pour pouvoir éventuellement rencontrer des hommes désirant participer à l'enquête. Concernant le vaccin, la majorité des participants savaient qu'il s'agissait de deux gouttes administrées par la bouche aux enfants (98%, taux élevé suggérant que la vaccination a été réellement effectuée) et que la poliomyélite était évitable par la vaccination (84%). Par contre, 35,7% des participants pensaient qu'il s'agissait d'une maladie curable. Ce résultat est retrouvé dans l'étude de Joseph (en 2011) avec 27,2% de croyances erronées sur la maladie. Notre étude et celle de Joseph ont toutes deux été menées dans une zone urbaine où les autorités ont mobilisé beaucoup de moyens de communication pouvant expliquer le niveau de connaissance sur la maladie et la vaccination des populations. La résidence en zone urbaine pourrait favoriser positivement les connaissances sur les maladies évitables par la vaccination [15].

Les principales sources d'information sur la connaissance des campagnes de vaccination étaient la radio (97,6%) suivi de l'information donnée par les agents vaccinateurs (72,4%). Dans plusieurs études, la radio de proximité a été jugée la plus fréquente source d'information pour la sensibilisation de masse [14, 16]. La radio de proximité est une source clé à utiliser lorsqu'on souhaite diffuser des informations de santé publique dans une zone où peu de personnes possèdent une télévision et où le taux d'alphabétisation est faible. D'ailleurs, plusieurs études mettent en évidence l'influence des médias et de l'information sur la participation des parents aux campagnes de masse [17]. Au Pakistan des enfants non vaccinés lors des campagnes de masse avaient des parents ayant un accès limité aux informations comme dans l'étude de Weiss [17]. Cette insuffisance d'information conduit à une mauvaise connaissance de la maladie et de la vaccination. Une autre étude pakistanaise avait montré que les connaissances au sujet de la poliomyélite étaient insuffisantes pour 38,8% des participants [12]. De plus les informations données par les agents vaccinateurs, dans une étude indienne ont joué un rôle très limité (7,2%) dans la connaissance de la maladie et le vaccin contrairement à nos résultats (72% d'information via les agents vaccinateurs). Nos résultats sont plus conformes au rôle attendu des agents vaccinateurs: ils sont censés être le moyen le plus efficace pour transmettre la bonne information. Ils sont choisis parmi la communauté et sont connus pour influencer la connaissance des populations locales par l'approche interpersonnelle pendant les activités de vaccination supplémentaires et surtout auprès des ménages [18].

En dépit d'une bonne connaissance sur la plupart des aspects de la maladie et du vaccin, plus de la moitié (61%) des participants avaient déclaré avoir subi l'influence négative de leur entourage. Beaucoup de rumeurs circulaient au sein de la population, la plus fréquente était une stérilité prétendue effet secondaire de la vaccination poliomyélite des enfants. Selon une étude ivoirienne, ces rumeurs sur la stérilité étaient un motif de refus fréquent de la vaccination par la communauté [14]. Dans notre étude, seulement 3% des parents interrogés avaient déclaré avoir refusé la vaccination. Un niveau scolaire très bas (non scolarisé ou n'ayant pas dépassé l'école primaire) était retrouvé. Les hommes étaient tous des commerçants et les femmes étaient des ménagères et habitaient dans les 2^e^, 3^e^ et 5^e^ arrondissements. Ces arrondissements constituent les quartiers les plus pauvres de la ville avec une forte densité de population et un faible accès à l'éducation. Les motifs de non vaccination se caractérisaient par la circulation des fausses rumeurs sur la vaccination. Ils croyaient tous en l'existence de substances nocives dans le vaccin qui pourraient provoquer des effets secondaires et avaient un manque de confiance sur la vaccination. Ces attitudes négatives vis-à-vis de la vaccination par la population générale ont déjà été mentionnées dans les études de Joseph, Murele et Khan, en Inde, au Nigéria et au Pakistan [12, 13, 18]. L'influence négative de l'entourage était souvent évoquée ainsi que la crainte sur la stérilité future de leurs enfants. De plus ces familles ayant déclaré refuser la vaccination se posaient la question sur les moyens déployés pour atteindre les enfants chez eux dans leur domicile alors qu'il y'avait d'autres priorités de santé.

Tous les ménages visités ont déclaré que leurs enfants ne possédaient pas un carnet de vaccination et 99% des vaccinations contre la poliomyélite ont eu lieu à domicile lors des activités de vaccination supplémentaires. L'absence de carnet est un frein majeur pour le suivi de la couverture vaccinale des enfants. C'est ainsi que dans une étude camerounaise, les enfants qui n'en possédaient pas étaient exclus [15]. Dans notre étude, cette absence de carnet n'a pas permis aux enquêteurs de vérifier les déclarations de vaccination contre la poliomyélite des enfants des participants. La vaccination complète et efficace des enfants n'a pas pu être vérifiée constituant un biais de classement majeur. Au Tchad, l'absence de carnet de vaccination présente un frein à la politique de santé publique visant l'éradication de la poliomyélite. En absence de preuve de vaccination, il est impossible de permettre une bonne stratégie d'éradication, car pas de suivi possible du protocole vaccinal. Au vu de cette enquête, les multiples campagnes organisées semblent avoir porté leur fruit, les parents bien informés comprennent la nécessité de la vaccination et des programmes de vaccination. Malgré la forte participation aux campagnes de vaccination contre la poliomyélite et une connaissance relativement bonne sur la maladie et le vaccin, les fausses rumeurs et les idées négatives circulaient encore au sein de la population comme dans nombreux pays d'Afrique. D'où la nécessité d'intégrer l'éducation pour la santé et une communication ciblée et adaptée dans le programme d'éradication de la poliomyélite. Les deux principales limites de l'étude sont: l'absence de carnet de vaccination. Les enquêteurs ont dû se contenter de la déclaration ce qui a pu entrainer un biais important dans les résultats; la participation volontaire de parents choisis au hasard dans les ménages ciblés, ce qui pourrait potentiellement engendrer un biais d'auto-sélection d'un membre de la famille plus favorable à la vaccination. Cette étude préliminaire a confirmé que l'absence de traçabilité de la vaccination ne permet pas d'évaluer correctement la politique vaccinale, car les résultats sont biaisés par un recueil uniquement déclaratif du statut vaccinal des enfants.

## Conclusion

Les résultats de cette étude ont révélé que les participants ont des connaissances sur la poliomyélite et la vaccination. Cependant, il existe encore des personnes réticentes à la vaccination et qui propagent des rumeurs influençant négativement leur entourage. Ces fausses rumeurs pourraient amener beaucoup de parents indécis à refuser la vaccination future de leurs enfants. Des interventions de communication et de sensibilisation ciblées et personnalisées devront continuer à être organisées auprès des parents. Enfin, doter les enfants d'un carnet de vaccination et le rendre obligatoire semblent être une action prioritaire pour mesurer la couverture vaccinale poliomyélite au Tchad.

### Etat des connaissances actuelles sur le sujet

- Refus de la vaccination de certaines populations;
- Les variations de couverture vaccinale en fonction des régions;
- Mise en œuvre de stratégies de vaccination.

### Contribution de notre étude à la connaissance

- Absence totale de carnet de vaccination;
- Population semble accepter la vaccination;
- Circulation des rumeurs négatives constitue un frein à la vaccination.

## Conflits d’intérêts

Les auteurs ne déclarent aucun conflit d’intérêts.

## References

[1] Global Polio Eradication Initiative Polio Eradication and Endgame Strategic Plan 2013-2018.

[2] Centers for Disease Control and Prevention (2012). Progress toward poliomyelitis eradication-Chad, January 2011-August 201. MMWR Morb Mortal Wkly Rep.

[3] Khan TM, Sahibzada MUK (2016). Challenges to health workers and their opinions about parents' refusal of oral polio vaccination in the Khyber Pakhtoon Khawa (KPK) province, Pakistan. Vaccine.

[4] Lee M, Hampton, Margaret Farrell, Alejandro Ramirez Gonzalez, Lisa Menning, Stephanie Shendale, Ian Lewis (2016). Abandon du vaccin antipoliomyélitique oral trivalent et introduction du vaccin antipoliomyélitique inactivé à l'échelle mondiale. Relevé épidémiologique hebdomadaire.

[5] Anya B-PM, Moturi E, Aschalew T, Carole Tevi-Benissan M, Akanmori BD, Poy AN (2016). Contribution of polio eradication initiative to strengthening routine immunization: Lessons learnt in the WHO African region. Vaccine.

[6] Ndiaye SM, Ahmed MA, Denson M, Craig AS, Kretsinger K, Cherif B (2014). Polio outbreak among nomads in Chad: outbreak response and lessons learned. J Infect Dis.

[7] Ministère de la santé publique (2011). Plan d'urgence pour l'interruption de la circulation du poliovirus sauvage au Tchad.

[8] Commune d'Abéché (2015). Plan de développement communal d'Abéché: rapport diagnostic.

[9] Institut National de la Statistique, des Études Économiques et Démographiques (INSEED) Deuxième recensement général de la population et de l'habitat (RGPH2, Septembre 2009).

[10] OCHA (2012). Profil du Ouaddai.

[11] Organisation Mondiale de La Sante (2015). Enquêtes de couvertures vaccinales par sondage en grappe: Manuel de référence.

[12] Khan MU, Ahmad A, Aqeel T, Salman S, Ibrahim Q, Idrees J (2015). Knowledge, attitudes and perceptions towards polio immunization among residents of two highly affected regions of Pakistan. BMC Public Health.

[13] Murele B, Vaz R, Gasasira A, Mkanda P, Erbeto T, Okeibunor J (2014). Vaccine perception among acceptors and non-acceptors in Sokoto State, Nigeria. Vaccine.

[14] Yao GHA, Aka LBN, Manouan NJM, Effi OA, Douba A, Zengbé-Acray P (2014). Connaissances et attitudes des organisations de la société civile à la mise en œuvre du Programme élargi de vaccination de routine en Côte d'Ivoire. Santé Publique.

[15] Nguefack F, Kobela M, Dongmo R, Tassadong C, Mah E, Kago I (2016). Connaissances, attitudes et pratiques des mères travailleuses vis-à-vis de la vaccination des enfants: exemple des revendeuses de vivres des zones de faible performance vaccinale. Health Sci Dis.

[16] Curry DW, Perry HB, Tirmizi SN, Goldstein AL, Lynch MC (2014). Assessing the effectiveness of house-to-house visits on routine oral polio immunization completion and tracking of defaulters. J Health Popul Nutr. juin.

[17] Weiss WM, Winch PJ, Burnham G (2009). Factors Associated with Missed Vaccination during Mass Immunization Campaigns. J Health Popul Nutr. juin.

[18] Joseph N, Subba S, Nelliyanil M, Kotian S, Haridath A, N K (2011). A study of the knowledge and attitude towards pulse polio immunization in semi urban areas of South India. Australas Med J.

